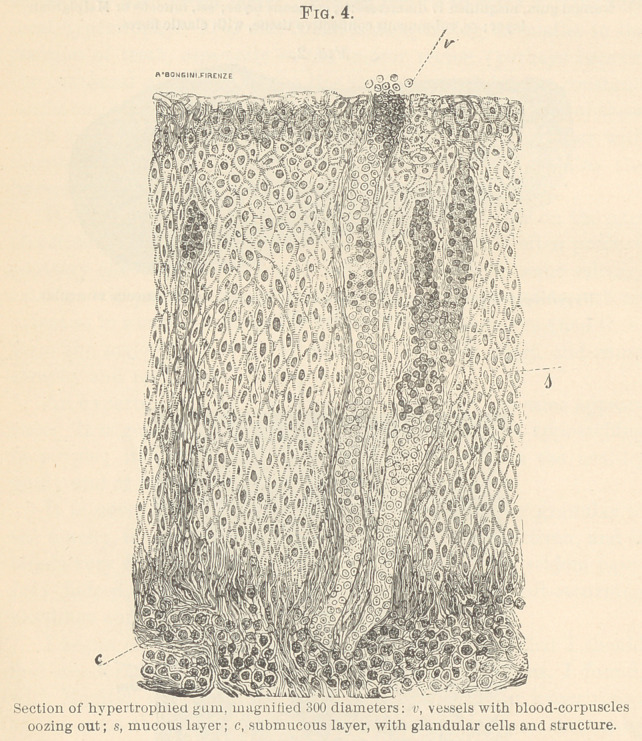# Hypertrophic Gingivitis: Histological Researches

**Published:** 1899-11

**Authors:** Luigi Arnone

**Affiliations:** Pisa, Italy


### Abstracts and Translations.

### HYPERTROPHIC GINGIVITIS: HISTOLOGICAL RESEARCHES.[1]

BY DR. LUIGI ARNONE, PISA, ITALY.

It is not of rare occurrence, in the practice of dentistry, to find the gums of some patients affected by the disease known as hypertrophic gingivitis; but very few practitioners have, till now, described this affection of the mucous membrane of the gums.

Magitot's studies are the latest which have been published on the subject. He describes this hypertrophy as simply a phenomenon of hypergenesis in the fundamental anatomical elements.

[1] Translated from L'Odontologia, xxii. year, No 6, Palermo, with the author's kind permission, by W. Dunn, D.D.S., Florence, Italy.

On the other hand, Dubois, in his "Treatise on Surgery," observes that this hypertrophy is often accompanied by dental anomalies, especially in the position of the teeth, and expresses the opinion that hypertrophy is most often due to the transformation of a fungoid state, owing to the diminution in the diameter of capillaries, by cicatrization, and fibrous organization of the fungoid body. On this point, however, I cannot agree with Dubois.

Many are the causes of hypertrophy of the gums. It is found in patients with green tartar. Broken teeth and roots will produce local hypertrophy. It is also found accompanying anomalies in the position of teeth, especially in the incisor region (perhaps because the continual movement of the lips produces a slight, but constant, mechanical irritation). Besides, in the irregular interstices of these teeth food and mucous secretions are apt to lodge, together with micro-organisms, which rapidly develop; and this explains the nauseating emanations from the mouths of these patients.

It must also be admitted that a predisposition exists on the part of patients to hypertrophy, since the same causes will often produce contrary effects on two different individuals. For in some salivary calculus will produce, as before stated, hypertrophy, and to such an extent as to sometimes cover the teeth; whereas in others (and these form the majority) it will produce the contrary effect, and cause atrophy and recession of the gums.

An hypertrophied gum will not always present the same appearance. It is generally found of a dark-red color, almost approaching to purple; its consistency is always more pasty than the healthy gum; and at times it is softer and semitransparent.

It is never smooth on the surface, but feels rather granular to the touch; it is easily detached from the necks of the teeth, and it bleeds very freely on the slightest provocation. It is seldom painful; indeed, the only troublesome features are the oft-returning bleedings and bad breath.

I began my researches with the preconceived idea that I should find neo-formations in these fungoid growths; whereas, I found, with Magitot, that in all and every form of atrophy or hypertrophy of the gums there is no histological difference between the healthy and diseased tissue; it is only an alteration in the relation between the different tissues.

In order to get a clear idea upon the modest researches I have been able to make, I have placed sections of normal gum tissue beside the pathological sections; these were collected from the dis-

Fig. 1.

Normal gum, magnified 17 diameters: *e*, corneous layer; *sm*, mucous or Malpighian layer; *co*, submucous connective tissue, with elastic fibres.

Fig. 2.

Hypertrophied gum, magnified 17 diameters: *v*, vessel; *e*, submucous muscular layer; *p*, Malpighian layer.

Fig. 3.

Section of normal gum, magnified 300 diameters: *e*, corneous layer; *cp*, capillaries; *sm*, papilla of the mucous layer; *co*, submucous connective tissue.

secting rooms of the University of Pisa, through the kindness of Dr. Bertelli.

A transverse section of the gum differs but little from a section of the skin in any other part of the body.

In fact, we find in Fig. 1 the corneous layer, the pellucid layer, the Malpighian layer, and the submucous layer.

Fig. 2 represents a section of hypertrophied gum, hardened in alcohol, and seen by low power. In this the alterations are: (1)

FIG. 4.

Section of hypertrophied gum, magnified 300 diameters: *v*, vessels with blood-corpuscles oozing out; *s*, mucous layer; *c*, submucous layer, with glandular cells and structure.

Weakening of the superficial layers; (2) loss of shape and irregular arrangement of the papillæ in the mucous layer; (3) dilatation of the blood-vessels, some of which open on the free edge of the gum point marked at *v* (labial aspect of the gum in the incisor region).

If we cast our eyes now on Fig. 4, an enlargement of 300 diameters of hypertrophied gum, we can see even better the weakening and thinning of the outer layers.

At *v* one can see blood-globules coming out of the free edge of the gum, through an open blood-vessel, some of them forming a clot near the opening. The cells of the mucous layer are larger and longer than the normal ones, and their nucleus three or four times the original size.

Whilst Fig. 3, with the same power, section of normal gum, shows distinctly the endothelium of blood-vessels, Fig. 4 will show the blood-vessels greatly distended, with their walls pressing against the Malpighian layer, the cells of which are flattened and compressed.

These sections were stained with hæmatoxylin (Weigert), but others treated with other stains have invariably displayed the same arrangement, the same exaggerated production of the usual constituting elements.

The continuation of the blood-vessel onto the free edge of the gum explains the frequent small hemorrhages met with in such cases, and which are so difficult to control or to stop quickly. The walls of the vessels, not being protected by other tissues, tear and fray with the greatest ease; the vessels then disgorge themselves, become flaccid, and after a while are closed again by a slight clot. But a slight increase in the blood-pressure, as in walking or lowering the head, or even in talking, forces out the clot, and the bleeding commences again.

With regard to remedies, I have tried every possible astringent, and even caustics, without result. Actual cautery does not always answer; indeed, at times the growth has increased after such remedies, probably because cauterization acts as an irritant.

The only remedy I have found efficacious is a free excision of the spongy or softened portion of the gum, and, after the surface has been left to bleed for a few minutes, to produce a fairly consistent eschar by actual cautery. The eschar will peel off in a day or two, leaving a healthy, granulating surface beneath, which will take the appearance of the gum after ten or twelve days.

During this time it is well to advise the patient to use alkaline solutions as mouth-washes, so as to neutralize any acidity of the saliva, and to keep the mouth clean; also prescribe disinfecting mouth-washes, three or four times a day; and a solution of carbolic acid, four per cent., to be kept in the mouth for a few minutes.

Therefore, on the strength of histological research, one may discard absolutely the idea that in hypertrophy of the gum, in the softening, in fungoid growths, there may be any neo-formations, even though a superficial examination, and the microscopic aspect, might lead one to suppose that these growths are a proliferation of the mucous membrane.

## Figures and Tables

**Fig. 1. f1:**
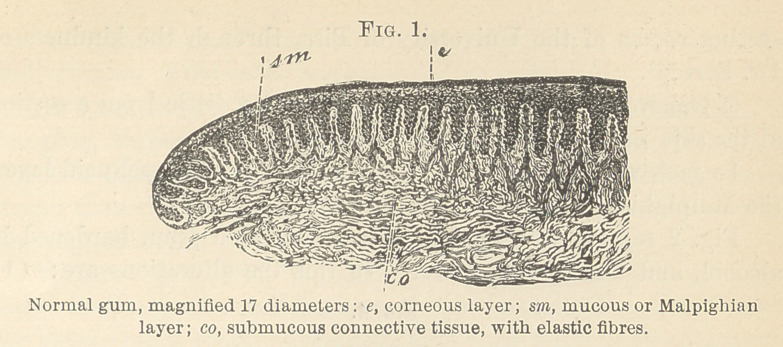


**Fig. 2. f2:**
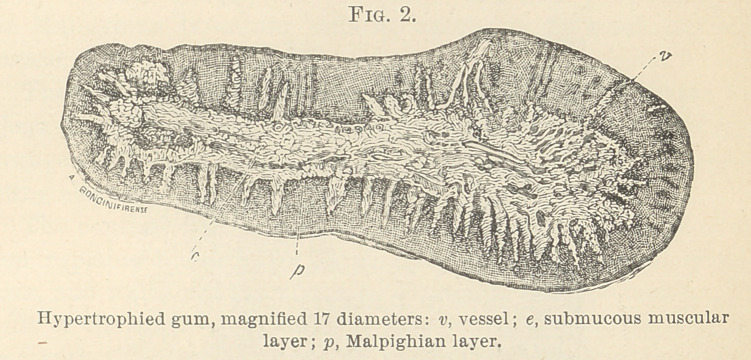


**Fig. 3. f3:**
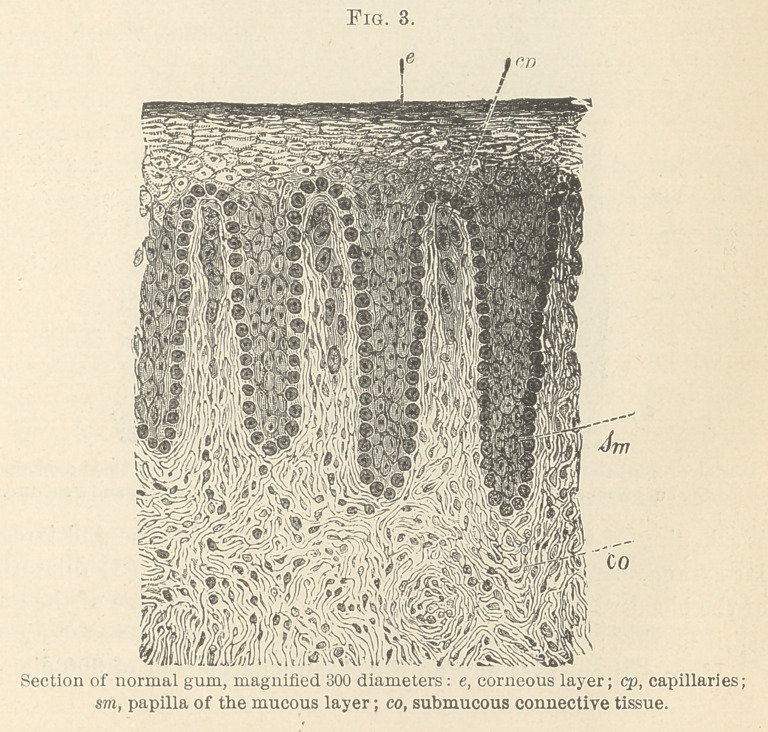


**Fig. 4. f4:**